# Validation for Brazilian Portuguese of the Eating Behavior Phenotypes Scale (EFCA): confirmatory factor analysis and psychometric properties

**DOI:** 10.20945/2359-4292-2024-0404

**Published:** 2025-08-04

**Authors:** Ronaldo José Pineda-Wieselberg, Andressa Heimbecher Soares, Thiago Fraga Napoli, Vanesa Erica Anger, Jesica Formoso, Maria Luciana Larrouyet Sarto, Nilza Maria Scalissi, João Eduardo Nunes Salles

**Affiliations:** 1 Departamento de Medicina Interna, Santa Casa de Misericórdia de São Paulo, São Paulo, SP, Brasil; 2 Centro Interdisciplinario de Investigaciones en Psicología Matemática y Experimental, Consejo Nacional de Investigaciones Científicas y Técnicas, Buenos Aires, Argentina; 3 Instituto de Biofísica Carlos Chagas Filho, Universidade Federal do Rio de Janeiro, Rio de Janeiro, RJ, Brasil

**Keywords:** Obesity, Precision medicine, Phenotype

## Abstract

**Objective:**

To validate the psychometric properties of the Eating Behavior Phenotypes Scale (EFCA) and to analyze the stability of the construct and its external validity in Brazilian Portuguese.

**Subjects and methods:**

A total of 206 adult participants completed a self-administered survey designed to identify eating behavior phenotypes. Confirmatory factor analysis was performed, and internal consistency was assessed using Cronbach’s alpha coefficient. Concurrent validity was evaluated through Pearson’s correlation between EFCA scores and body mass index. Translation involved independent forward translation from Argentinian Spanish to Brazilian Portuguese, followed by back-translation from Brazilian Portuguese to Spanish. The Brazilian Portuguese version was administered following 100% agreement between the versions.

**Results:**

The EFCA and its subscales in Brazilian Portuguese showed acceptable internal consistency (α = 0.83).

**Conclusion:**

Confirmatory factor analysis indicated a good fit of the data to the proposed structure. No statistically significant correlation was found between the body mass index and each subscale or the total scale score. The translation and back-translation process yielded less than a 5% discrepancy between the versions.

## INTRODUCTION

The limited long-term efficacy of obesity treatments necessitates the identification of eating behavior phenotypes and sub-phenotypes. These serve as mediators between an individual’s genotype and the process of weight gain throughout the life cycle (^1^-^3^). Multiple dimensions of eating behavior shape specific patterns, including the use of food for emotional coping (emotional or stress-related eating), increased sensitivity to pleasurable or indulgent foods (hedonic eating), loss of control over caloric intake (compulsiveness or disinhibition), and a lack of moderation in consumption (hyperphagia) (^4^-^7^). As these eating styles consolidate, they form clusters of eating behavior phenotypes that significantly determine individual variation in caloric intake self-regulation and, consequently, the potential for weight gain. Research interest in eating behavior phenotypes among individuals with overweight and obesity has increased, presenting opportunities for both pharmacological and non-pharmacological interventions grounded in precision medicine (^8,9^).

This study evaluated the psychometric properties of the Eating Behavior Phenotypes Scale (EFCA) and its validation for Brazilian Portuguese. Originally designed and validated in Argentinian Spanish (^10^), the scale characterizes eating behavior phenotypes, focusing on internal consistency, criterion validity, and construct validity, as indicated by prior research.

This study aimed to validate the psychometric properties of the Eating Behavior Phenotypes Scale (EFCA) and to analyze the stability of the construct and its external validity in Brazilian Portuguese

## SUBJECTS AND METHODS

### Participants

A sample of 206 Brazilian Portuguese-speaking individuals aged 18 or older was randomly selected from participants recruited anonymously via social media (Instagram and Facebook) in 2024. The sample size was estimated according to recommendations in the literature (^11^-^13^), which suggest a ratio of ten participants per item and a minimum of approximately 200 participants. After providing their informed consent, the participants filled out a self-administered electronic form that included demographic data and self-reported height and weight. It also included the Brazilian Portuguese version of the EFCA. The participants received no financial compensation for their participation. The study adhered to all guidelines outlined in the Declaration of Helsinki and was approved by the Research Ethics Committee of the *Irmandade da Santa Casa de Misericórdia de São Paulo* (CAAE no. 81977624.7.0000.5479).

### The Eating Behavior Phenotypes Scale

The EFCA comprises 16 items on a 5-point Likert scale (ranging from “never” to “always”), each describing a specific attitude towards food. Participants completing the scale must indicate how frequently they express the specific attitude, and the different eating behavior traits form five subscales or eating behavior sub-phenotypes are defined as follows:

- Disorganized: skipping at least one main meal or having an inter-meal period longer than 5 hours.
- Hedonic: a desire to eat triggered by sensory (visual and olfactory) and/or cognitive stimuli.
- Compulsive: rapid and excessive food intake in short periods.
- Emotional: use of food as a coping strategy triggered by negative emotions (anxiety, boredom, loneliness, fear, anger, sadness, and/or fatigue) or frequent small snacks between main meals.
- Hyperphagic: consumption of excessive portions or more than one serving in a single meal.

The EFCA’s original factor structure demonstrated a good fit, with factor loadings above 0.40 in all cases. Cronbach’s alpha coefficient indicated an acceptable reliability of 0.86 for the total scale, with subscales ranging from 0.73 to 0.88 (sub-phenotypes: grazer/emotional: α = 0.88; hyperphagic: α = 0.84; hedonic: α = 0.73; disorganized: α = 0.73; compulsive: α = 0.83). The total score is calculated by summing the response to each item (1 = never to 5 = always, except for question 9, which is reverse-scored).

### Anthropometry

The participants’ reported weight (kg) and height (cm) were used to calculate the body mass index (BMI). Individuals were classified as underweight, normal weight, overweight, or obese according to the cutoff points provided by the *Associação Brasileira para o Estudo da Obesidade e Síndrome Metabólica* (Abeso), the *Sociedade Brasileira de Endocrinologia e Metabologia* (SBEM), and the Brazilian Ministry of Health.

### Statistical analyses

To assess criterion validity, Pearson’s correlation coefficients were calculated between the scale — both total values and subscales — and the participants’ BMI. Since the EFCA designed to assess unhealthy eating styles, we used the BMI as the external criterion, mirroring the approach of the original scale. Reliability was evaluated through internal consistency, measuring Cronbach’s alpha coefficient for the total scale and each of its subscales. Additionally, the structural stability described by Anger and cols. was verified through confirmatory factor analysis (^10^), employing the maximum likelihood estimation method. This analysis was conducted using RStudio statistical software.

## RESULTS

The EFCA was validated with 206 participants surveyed between September 23, 2024, and September 25, 2024. The participants’ ages ranged from 18 to 73 years, with 99 identifying as men and 107 as women. **Table 1** summarizes the responses and characteristics of the evaluated population.

**Table 1:** Populational evaluation of the sample

	n (%)	Mean	SD	Minimum	Maximum
Sex					
Female	107 (52)				
Male	99 (48)				
Age (years)		47.28	16.02	18	73
BMI (kg/m^2^)		30.03	6.26	17.3	44.5
Eating behavior phenotypes scale					
Total		41.24	9.32	21	77
Disorganized		7.32	3.03	3	15
Compulsive		4.99	2.14	2	10
Hedonic		12.37	3.13	5	20
Emotional		9.78	3.57	4	20
Hyperphagic		6.77	2.19	3	15

SD: standard deviation; BMI: body mass index.

**Figure 1:**
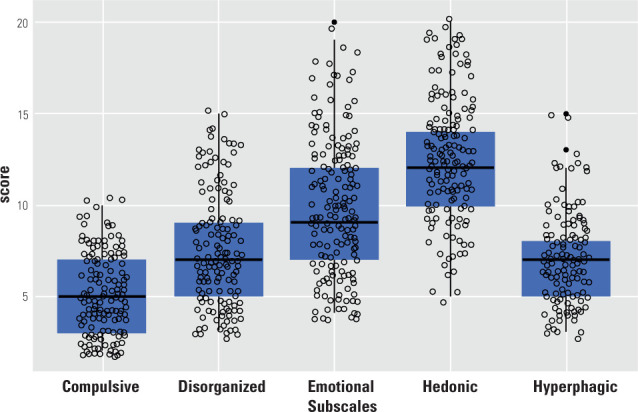
Result dispersion for each subscale of the Eating Behavior Phenotypes Scale.

The applied version was obtained after a certified translation of the original scale from Spanish to Brazilian Portuguese and a certified back-translation by independent translators without prior access to each other’s work. These versions were compared, achieving 100% agreement; the Brazilian Portuguese version was consequently applied. **Table 2** lists the Brazilian Portuguese version of the scale, while **Table 3** outlines the scoring system for EFCA and its subscales. **Figure 1** shows the distribution of scores across each subscale.

**Table 2:** Portuguese-translated version of the Eating Behavior Phenotypes Scale applied to the sample

Eating Behavior Phenotypes Scale
Question
1. *Eu como até ficar muito cheio* 2. *Acalmo as minhas emoções com comida* 3. *Peço mais comida quando termino meu prato* 4. *Tenho o hábito de petiscar (petiscar = fazer pequenas refeições entre as refeições principais – café da manhã, almoço, café da tarde e jantar – sem medir a quantidade do que se come)* 5. *Quando começo a comer algo que gosto muito, tenho dificuldade em parar* 6. *Costumo comer mais de um prato nas refeições principais* 7. *Lanches entre as refeições devido à ansiedade, tédio, solidão, medo, raiva, tristeza e/ou cansaço* 8. *Sinto-me tentado a comer quando vejo/cheiro comida que gosto e/ou quando passo por um quiosque, uma padaria, uma pizzaria ou um estabelecimento de* fast food 9. Tomo café da manhã todos os dias* 10. *Como nos momentos em que estou: entediado, ansioso, nervoso, triste, cansado, irritado e solitário* 11. *Pulo algumas – ou pelo menos uma – das refeições principais (café da manhã, almoço, café da tarde ou jantar)* 12. *Quando me deparo com uma comida que gosto muito, mesmo sem sentir fome, acabo comendo* 13. *Como muita comida em pouco tempo* 14. *Quando como algo que gosto, finalizo toda a porção* 15. *Quando como algo que gosto muito, como muito rápido* 16. *Passo mais de 5 horas por dia sem comer*

* Question 9 scoring must be reversed.

**Table 3:** The Eating Behavior Phenotypes Scale total and subscale scoring system

Scale	Low	Medium	High
Total	16-37	38-48	≥ 49
Disorganized (questions 9, 11, and 16)	≤ 4	5 and 6	≥ 7
Hedonic (questions 5, 8, 12, and 14)	≤ 11	12-14	≥ 15
Compulsive (questions 13 and 15)	≤ 3	4-6	≥ 7
Emotional (questions 2, 4, 7, and 10)	≤ 8	9-12	≥ 13
Hyperphagic (questions 1, 3, and 6)	≤ 5	6-8	≥ 9

### Validation procedures

#### Internal consistency

To evaluate internal consistency, Cronbach’s alpha was computed for the total scale and for each subscale. The overall result showed an alpha of 0.83, with a 95% confidence interval between 0.80 and 0.86, suggesting good internal consistency. This value exceeds the acceptable threshold of 0.70, demonstrating that the scale items reliably measure the proposed constructs.

### Subscale results

#### Disorganized

The alpha coefficient was 0.75, with the highest correlations observed between questions 9 and 11, indicating a strong correlation with the factor. Question 16 exhibited a lower correlation, suggesting a greater variability in responses associated with this item.

#### Compulsive eating

The alpha coefficient was 0.79, with a strong correlation between questions 13 and 15 (r = 0.91), demonstrating that both items adequately represent compulsive eating behavior.

#### Hedonic eating

The alpha coefficient was 0.71, the lowest among the subscales. Question 14 showed a weak correlation (r = 0.289), indicating that this item may not adequately represent the hedonic eating factor, potentially due to issues in participant interpretation.

#### Hyperphagia

The alpha coefficient was 0.76, with robust correlations between questions 1, 3, and 6, suggesting that the scale reliably measures hyperphagia.

#### Emotional

The alpha coefficient was 0.83, indicating good internal consistency, with high correlations between questions 2, 7, and 10 (r = 0.84).

### Factorial analyses

#### Exploratory factor analysis

Bartlett’s test of sphericity was conducted to assess data suitability for factor analysis, yielding a significant value (p < 0.001) and indicating sufficient correlation between items for factor analysis. The Kaiser-Meyer-Olkin test, with an overall value of 0.83, confirmed sample adequacy for analysis. A parallel analysis revealed that five factors accounted for 57% of the data variance, confirming the scale’s multidimensional structure. However, item 14 had a low factor loading (0.29), suggesting it may not adequately represent the hedonic construct in the Portuguese version.

#### Confirmatory factor analysis

**Figure 2:**
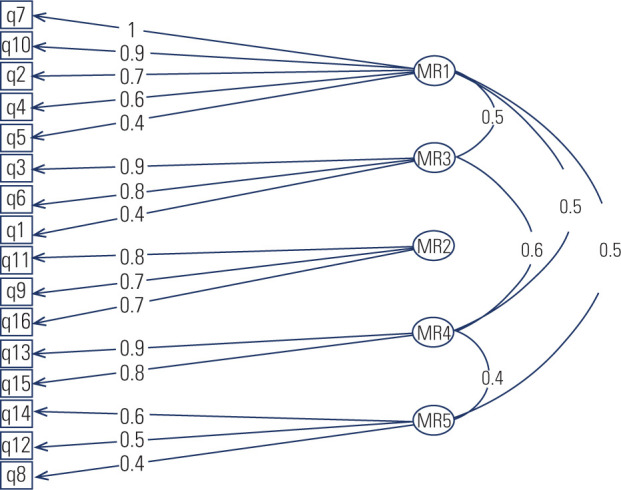
Factorial loading for the confirmatory factor analysis. A *p-*value < 0.001 was considered in all cases.

Based on exploratory factor analysis results, a five-factor model showed acceptable fit indices, with an internal factor coefficient of 0.924 and a root mean square error of approximation of 0.070, indicating a good fit. However, item 14’s poor fit to the hedonic factor underscores the need for its revision or exclusion from the scale. **Figure 2** shows the factor loadings from the confirmatory factor analysis.

### External validation with body mass index

The BMI was included as an external validation criterion to assess the concurrent validity of the EFCA. Nevertheless, the correlation between BMI and the total score was very low, as determined by Pearson’s correlation, with a coefficient of 0.0048 and p = 0.9448. This suggests that BMI was not a good predictor of the behavioral dimensions captured by the EFCA in this application.

## DISCUSSION

These findings align with the results of the original EFCA validation study, which reported correlations between BMI and subscales ranging from moderate to significant but varying across sub-phenotypes (^14^). In our study, however, BMI did not demonstrate a significant correlation. In our application, the BMI data were self-reported based on respondents’ weight and height, which may have introduced significant bias into the evaluation.

Other reasons for the lack of correlation between BMI and the EFCA may include that BMI does not capture daily eating behavior, non-behavioral factors that affect BMI, the temporal nature of BMI, with potential behavioral changes not yet reflected in BMI, and the possibility that patients may not perceive their behaviors accurately. Therefore, it is important to consider other markers as potentially valid for future evaluations.

### Identified implications and issues

Eating behavior is influenced by emotional, psychological, and social factors, not directly linked to body weight or BMI. Thus, the scale could be used to identify eating behavior phenotypes before the onset of obesity, facilitating early and preventive treatment.

Additionally, the analyses indicate that the scale is well-suited to capture eating behavior phenotypes, such as compulsive and hedonic behavior, but future validations could benefit from an external criterion more closely related to the measured behaviors, such as the frequency of binge eating episodes or the presence of specific eating disorders.

When evaluating Question 14 as potentially the least appropriate, it is important to consider the cultural context in which the questionnaire is administered. For instance, in Brazilian culture, leaving food on one’s plate is generally unacceptable. Some establishments may even charge fees for customers who do so, although this practice is considered illegal in Brazil (^15^). Therefore, it is possible that item 14 was influenced by the cultural norm of always finishing one’s portion, even when the individual is already satisfied.

In conclusion, the Eating Behavior Phenotypes Scale demonstrated good internal consistency and psychometric validity, making it a valuable tool for assessing various eating behavior phenotypes. However, its external validation with body mass index was limited, indicating that future research should consider alternative external criteria, such as emotional factors or specific eating disorders, to evaluate the scale’s validity more effectively.
